# The Guiding Principles of Treatment in Excessive Corpulence

**Published:** 1888-04

**Authors:** George H. Rohe


### *THE GUIDING PRINCIPLES OF TREATMENT IN EXCESSIVE CORPULENCE.*

By GEORGE H. ROHE, M. D.

The corpulency of alcohol and beer drinkers is well known. It is likely that the depression of nervous excitability, due to the constant use of alcohol, is partly responsible for the fat-accumulation, although the alcohol in ardent spirits, and in the case of beer, the carbohydrates additionally present, acting as respiratory food, save a portion of the fat in the food from oxidation, and permit its deposit as fat-tissue. The Banting diet consists in a large excess of proteids, and great diminution, almost to extinction from the bill of fare, of fat and carbo-hydrates. On the theory that proteids alone are muscle-builders, while fats and carbo-hydrates are fat-forming foods, the Banting diet was correct, but the evils of a strict adherence to it were manifest before the recent physiological researches demonstrated the fallacies of the theory. There can be no doubt that Bantingism will reduce excessive fatness, but it also decreases the proteid constituents of the tissues, and may thus cause dangerous and even fatal consequences. It is frequently impossible to arrest the rapid waste of muscular tissue under the Banting diet, and the individual rapidly declines in health, even if the diet is withdrawn and the patient put on liberal allowance.

On account of the evil results which were frequently noticed as following the strict Banting diet, this has been modified in various ways. Vogel, who introduced the method in Germany, allowed a larger proportion of carbo-hydrates, while Immermann proposes to interrupt the treatment occasionally, and allow the patients to resume their habitual diet for a period. On the other hand, Cantani, the eminent Italian clinician, insists even more strongly than Banting, upon the rigid exclusion of fats and carbo-hydrates. Beer, spirits and the stronger wines are restricted or excluded from all dietaries for corpulence. Light acid wines are alone allowed.

In the last five years, two new methods, deviating in a rational direction from Bantingism, have attracted the attention of the profession. The first is that of Professor Ebstein, of Göttingen, who proposes a reduction of proteids and carbo-hydrates and an increase of fat. The philosophical reasoning of Ebstein claims careful consideration, since he bases it upon acknowledged physiological facts. He claims that less fat is deposited if there is much fat in the food than if the food contained an equivalent quantity of carbo-hydrates. Practical experience sustains this claim, as well as the further one that a smaller proportion of proteids suffices than is generally assumed. But an important point insisted upon by Ebstein is usually overlooked by his followers, namely, that active muscular exercise assists in the destruction of fats.

Oertel's method, which has also many earnest advocates, approaches, in its dietetic prescriptions, very closely, the Banting system, as will be shown presently by a comparison of the different classes of food-materials in the various plans; but an important element in Oertel's regimen is the extremely small quantity of fluids permitted. This is not original with Oertel, as Dancel had already, in 1863, insisted upon its importance.

Professor Kisch, of Prague, who is resident physician at the famous sulphate-of-soda springs of Marienbad, has had a very large experience in the treatment of corpulence. He prescribes the waters (which act as a purgative), advises active exercise, and regulates the diet. Doubtless, good results follow this treatment, for it is based upon rational principles. The treatment by cold baths and exercise is also successfully applied for the relief of this condition by Winternitz, of Vienna.

Directly contrary to the advice of Oertel to reduce the fluid allowance of corpulent patients, See, of Paris, largely increases it. "Let your patients drink—drink all the time," is the advice he gives. He thinks tea is the best beverage, and that it should be consumed in large quantities. This practice is based upon the fact that free drinking of water promotes tissue-metabolism, and hence increases the oxidation of the fat, as pointed out by Landois.

There is abundant testimony that all these methods are efficient for the reduction of corpulence. For comparison, the following calculations of the amounts of the various food-stuffs in the Banting, Ebstein and Oertel diets are given:

| | Banting. | Ebstein. | Oertel. |
|---|---|---|---|
| Proteids . . . . | 170-180 grms. | 105 grms. | 156-170 grms. |
| Fats . . . . . . | 7.5 grms. | 60-100 grms. | 22-43 grms. |
| Carbo-hydrates . | 80-85 grms. | 45-50 grms. | 71-114 grms. |

Landois recommends a gradual and equable reduction of the entire bill of fare, with active exercise in the open air, purgation, cool bathing, stimulation of the skin, restricted hours of sleep, light clothing, and free consumption of liquids. There is reason to believe that such a regimen is more efficient and safer, even if not so rapid in its action, than a plan which depends principally upon the withdrawal of one or other class of food-materials.—*Philadelphia Medical Times.*

